# Supplementary material for: Maternal death surveillance and response system evaluation in Makonde District, Zimbabwe, 2021
Source: PLoS One. 2024 Jul 1;19(7):e0301929. doi: 10.1371/journal.pone.0301929 (PMC11216583; doi:10.1371/journal.pone.0301929)
Supplement: S1 File — (DOCX) [file pone.0301929.s002.docx]

**Annexe 1: Consent Form**

**QUESTIONAIRE NUMBER [ ]**

**Evaluation of the MDSR system in Makonde District, 2021**

**Consent Form for Study Participants**

**Principal Investigator**: Dr Tsitsi B. Makanyanga

**Mobile Number**: 00263 772768402

**Introduction**

My name is Tsitsi Brenda Makanyanga. I am a student at the University of Zimbabwe studying Public Health. I am evaluating the Maternal Death Surveillance and Response system in Makonde District and I would like you to participate. Your responses are confidential and information collected will be used to improve the Maternal Death Surveillance and Response system in Makonde District. You will be assigned a number called a participant ID to identify you in this study in order to protect your identity. Participating in this study is voluntary and you can withdraw consent at any time, you can also skip certain questions that you are not comfortable with. There are no incentives for participating in this study.

If you consent to participating in this study please sign below;

Participant’s signature……………………………………. Date…………………….

Investigator’s signature………………………………….... Date……………………..

Witness’s signature……………………………………….. Date………………………

**Annex 2: Health Worker Questionnaire**

Name of Health Facility...........................................

| **No** | **Variable** | | **Response** | | |
| --- | --- | --- | --- | --- | --- |
| **DEMOGRAPHIC CHARACTERISTICS** | | | | | |
| 1 | Sex | | Male [ ]  Female[ ] | | |
| 2 | Designation | | RGN  PCN  Midwife  Government Medical Officer | | |
| 3 | Year of attaining post-basic qualification | |  | | |
| 4 | Period of operating the surveillance system | |  | | |
| 5 | Year of joining current institution | |  | | |
| 6 | Setting of facility currently employed | | Rural health centre  Rural hospital  Private clinic  Council clinic  Provincial referral hospital | | |
| **KNOWLEDGE OF THE TB SURVEILLANCE** | | | | | |
| 7 | Did you receive any training in MDSR | | | | Yes [ ] No [ ] |
| 8. If yes, what type of training did you receive?  On the job training [ ]  Workshop [ ]  Mentorship [ ]  Post basic qualification [ ] | | | | | |
| 9 | What is the MDSR? | | | | Correct [ ]  Incorrect [ ]  Don’t know [ ] |
| 10 | What are the objectives of the MDSR? | | | | Correct [ ]  Incorrect [ ]  Don’t know [ ] |
| 11 | Define a woman of reproductive age | | | | Correct [ ]  Incorrect [ ]  Don’t know [ ] |
| 12 | Define a maternal death | | | | Correct [ ]  Incorrect [ ]  Don’t know [ ] |
| 13 | What is the time limit for notification of a maternal death to the next level by phone? | | | | Correct [ ]  Incorrect [ ]  Don’t know [ ] |
| 14 | Do you receive any feedback on the MDSR data that you submit? | | | | Yes [ ]  No [ ] |
| 15 If yes, in what form is the feedback?  Reports [ ]  Oral briefings [ ] | | | | | |
| 14 |  | | | |  |
| 14 | List the M&E tools that are updated following a maternal death. | | | | Correct [ ]  Incorrect [ ]  Don’t know [ ] |
| **USEFULNESS** | | | | | |
| 15 | Do you analyse maternal mortality trends? | | Yes [ ]  No [ ] | | |
| 16 If yes who analyses the information?.......................................................................................... | | | | | |
| 17 | Do you use data generated from the MDSR? | | Yes [ ]  No [ ] | | |
| 18 If yes, what is the data used for? (**Ask for maternal audit meeting minutes and any documentation with recommendations and public health actions that were implemented in response to MDSR).** | | | | | |
| 19 | Do you have meetings to discuss maternal deaths? | | Yes[ ]  No[ ] | | |
| 20 If yes, when was the last time that you held such a meeting? (**Verify with the maternal, neonatal and child health focal person)**  < 1 month ago [ ]  >1 months ago [ ]  Can’t remember [ ]  Minutes Available[ ]  Minutes not available[ ] | | | | | |
| 21 What are some of the recommendations from the audit, that you have ever received or implemented?........................................................................................................................  ……………………………………………………………………………………………..  …………………………………………………………………………………………………  …………………………………………………………………………………………………… | | | | | |
| 22 | Do the public health actions recommended by MDSR improve maternal health care? | | Yes [ ]  No [ ] | | |
| 23 | When was the last time that you received the feedback? | | < 1 month ago [ ]  >1 months ago [ ]  More than 3 months ago [ ] | | |
| 24 | How does the MDSR impact on how you provide maternal health services? | | Positively [ ]  Negatively [ ]  Indifferent [ ] | | |
| 25 | Do you think the MDSR system is useful? | | Yes [ ]  No [ ]  Not Sure [ ] | | |
| 26 If yes, please give reasons…………………………………………………………………………..  ……………………………………………………………………………………………………..  If no, please give reasons.  Data is not being utilized [ ]  No feed-back is given to health centers [ ]  Other, specify………………………………………………………………………………………..  …………………………………………………………………………………………………………… | | | | | |
| **SIMPLICITY** | | | | | |
| 27 | Have you ever filled a MDNF? | | Yes [ ]  No [ ] | | |
| 28 | How long does it take to fill in a MDNF? | |  | | |
| 29 | Have you ever had challenges in in any stage of the notification process? | | Yes [ ]  No [ ] | | |
| 30 If Yes, what are the challenges?  Other (please specify) ………………………………………………………………………………. | | | | | |
| 31 | Do you have problems with filling in the MDNF? | | Yes [ ]  No [ ] | | |
| 32 If yes, specify the difficult areas (**give copy of MDNF for respondent to indicate section).**  …………………………………………………………………….. | | | | | |
| 33 | Are there times when you fail to complete the MDNF? | | Yes [ ]  No [ ] | | |
| 34 If yes what are the reasons for the failure to complete the MDNF?  No time to complete the form [ ]  Work overload [ ]  Not part of my job description [ ]  Difficulties in accessing information [ ]  Not interested [ ]  Other, specify……………………………………………… | | | | | |
| 35 | | Are you confident to fill in a MDNF when need arises? | | Yes [ ]  No [ ] | |
| 36 If no specify the reasons? ........................................................................................................... | | | | | |
| **STABILITY** | | | | | |
| 37 | What data storage method do you use for the MDSR information? | | Electronic Medical Records[ ]  Paper based records[ ]  Both [ ] | | |
| 38 | What communication system devices are present at your institution? | | Telephone [ ]  Fax [ ]  Email [ ]  Postal Services [ ] | | |
| 39 | | Are the MDNF readily available when you want to use them? | | Yes [ ]  No [ ] | |
| 40 | | Have you ever had stockouts of MDNFs? | |  | |
| **ACCEPTABILITY** | | | | | |
| 41 | Does the MDSR affect how you provide maternal health service? | | Time consuming [ ]  Not time consuming [ ]  Indifferent [ ] | | |
| 42 | Do you feel it is your duty to fill the MDNF? | | Yes [ ]  No [ ] | | |
| 43 If no, whose duty is it? | | | | | |
| 44 | Have you ever failed to complete The MDNF? | | Yes [ ]  No [ ] | | |
| 45 If yes, what were the reasons for failing to compile data? | | | | | |
| 46 | Are you willing to continue participating in the MDSR? | | Yes [ ]  No [ ] | | |
| **TIMELINESS** | | | | | |
| 47 | Do you report deaths to the next level within the stipulated time? | | Yes [ ]  No [ ] | | |
| 48 If yes, state the time limit……………………………………………………. | | | | | |
| 49 | Do you fill in the MDNF within the stipulated timelines? | | Yes [ ]  No [ ] | | |
| 50 If yes, what is the time limit?................................................................................ | | | | | |
| 51 | After how long do you sit for a maternal audit following a death? | | 1 week  1 month  2 months  No specified period | | |
| 52 | Do you have any constraints in the operation of the surveillance system? | | Yes [ ]  No[ ] | | |
| 53 If yes, what are the reasons?………………………………………………………………………..  ………………………………………………………………………………………………………… | | | | | |
| 54 | What would you recommend for the improvement of the MDSR? | | Training on surveillance [ ]  Better communication system. [ ]  Increase in the staff compliment. [ ]  Getting regular feedback from the provincial level [ ]  Other (please specify | | |
