# Supplementary material for: Maternal death surveillance and response system evaluation in Makonde District, Zimbabwe, 2021
Source: PLoS One. 2024 Jul 1;19(7):e0301929. doi: 10.1371/journal.pone.0301929 (PMC11216583; doi:10.1371/journal.pone.0301929)
Supplement: Attachment — Submitted filename: Reviewers Comments.docx [file pone.0301929.s003.docx]

**Abstract**

**Introduction**

1. Your introductory statement “…with 810 women dying every day from preventable causes related to pregnancy” refers to figure as of 2017, five years behind. This (2017) should be refenced in the statement. Alternatively, the authors should revise the statement to report the current figures of maternal mortality globally and in sub-Saharan Africa and then in Zimbabwe.
2. The statement “Globally the maternal mortality ratio is declining however the average rate of decline is not fast enough to achieve SDG target of 70 maternal deaths per 100000 live births” is similar to the statement “The United Nations Sustainable Development Goal target 3.1 aims at reducing global maternal mortality ratio to less than 70 per 100 000 live births by 2030…” and conveys the same meaning. I suggest you consider merging the two.
3. The authors referenced “the Centre for Disease Control” which collaborated with the World Health Organization. Could you be specific about the particular Centre for Disease Control? We have so many Centres for Disease Control who could partner WHO to implement programmes in LMIC.
4. The authors explained that the “Maternal Death Surveillance and Response System (MDSR)” is what is partially being evaluated in Makonde District. Perhaps, the authors could be much clearer on the motivation for choosing the Makonde District. Although the authors explained that 62% of maternal death were notified and audited which presumably is low for them, this figure is left standing alone could rather be an impressive figure if compared contextually. Perhaps, it could be compared with the figures of other districts who implemented the “MDSR” to show a compelling reason for wanting to know the situation in the Makonde District in particular.
5. Also, note that “MDSR” used as an abbreviation in the manuscript does not exactly represent the “Maternal Death Surveillance and Response System (MDSR). Probably MDSRS. Please check and revise appropriately.
6. **General Comment** - The introductory aspects is generally scanty and does not address the topic area in literature, rational for the choice of study area and purpose of study, adequately.

**Study setting**

1. The authors gave statistics of antenatal care attendance in the district of study to include one visit etc. Again, we are not told what percentage made 8+ visits and if none, indicate so to put the reference data into proper perspective, especially as the WHO was part of the implementation of the MDSR.
2. The authors also left out health facility delivery percentages from the Makonde District, although they attempted to explain that health facilities existed widely with capabilities and with one referral centre – the Chinhoyi Provincial Hospital. Please add facility delivery figures of the district. It will be useful in your discussion.

**Maternal Death Surveillance and Response System in Zimbabwe**

1. **The statement** “…. health facilities and communities should include zero reporting in their routine reports” is not clear. Perhaps, you could explain what you mean by zero reporting.
2. *Fig 1: Flow of information in a Maternal Death Surveillance and Response System in Zimbabwe* is a good representation of information flow relative to the surveillance system. However, Mission Hospital/ Local Authorities is left standing alone with no link to source of information as though it is the final generator of information. It is not clear if the diagram is adopted or adapted or the original creation of the authors. Perhaps the authors could consider creating a link of information from the community to the mission hospital/local authority, since the community is the original and last source of maternal data.
3. Also, I don’t find an in-text reference to figure 1. which makes the figure appear not useful in the manuscript, but that is not the case. Please reference the figure in the write up appropriately.

**Study design**

1. “The updated CDC guidelines for 163 Surveillance System Evaluation” … was the updated version utilized in the Makonde District surveillance, otherwise, the authors may have to explain why they chose the updated version of CDC guidelines.
2. What is “T5” registers?

**Sample size and data collection**

1. **“A minimum sample size of 61 was calculated”.** This does not marry well with “we interviewed five doctors and forty nurses from the provincial hospital, two doctors and six nurses from the rural hospital, one nurse and one doctor from the private maternity clinic, ten nurses from the urban clinics and fourteen nurses from the rural clinics”. Clarity is necessary. Your sample calculation of the mortality record for desk top review must stand out clear from your choice of health care professionals for interviews.
2. Could the authors share the data collection tools?

**Ethical consideration**

1. Perhaps the authors could quote the approval code or number from the Mashonaland West Provincial Ethics Committee.

**Results**

1. Only 41% were midwives, although a large portion of the health professionals interviewed were nurses. The expectation is that midwives should be the main target group for interview in matters of maternal deaths and audit. The authors need to explain further why this was not the case. Perhaps numbers and availability and willingness, to add meaning to the results.
2. The writing of the results should be re-organised to give a good flow. For example, “Overall, 61 (77.2%) of the 248 healthcare workers displayed good knowledge of the MDSR system. Table 3 shows the results” should be the opening paragraph of that section and followed by other relevant portions of the results which the authors intend to communicate rather than make this statement in the ending paragraphs.
3. “Assuming a nurse’s salary of $400 USD and a distance of 120 km of the furthest health facility to the district hospital” what is the need for introducing a nurse’s salary in the cost component of the results write up? It appears tangential to the study discourse. In any case, the entire idea of cost does not add value to the study objective. It can be removed without a significant damage to the study results.

**Discussion**

1. Generally good-
2. The authors made a statement that “Healthcare workers in Makonde District presumed the MDSR system to be useful as they utilised the information generated from audits to improve maternal health service delivery and monitor maternal mortality” yet, the district doesn’t seem to achieve what is expected. Could there be some conflicting reasons??? This needs to come out clearly in the discussion.

**Limitation of study**

1. Although the study is useful, it is limited in many ways. For example, not targeting midwives more in the study, given they are the professional grouping mandated to handle pregnant women limits the validity of information obtained from the health workers. This has to be stated in the manuscript.

**Conclusion and Recommendations**

1. Your results show 87.3% know the importance of the tool, 91% know the timeliness importance, 97% thinks the system is useful, 88.6% are willing to continue with the surveillance system yet **“lack of motivation” and “training gap”** appeared in your conclusion? Readers will not understand this. The authors need to revise their conclusion based on the study results.
2. The authors rather gave general recommendation including training requirement of staff and also recommended the need for ambulance services to be effectively provided. I don’t find these as borne out of the study results/discussion, especially as the surveillance process did not involve ambulance system and the results suggest the staff had good knowledge of the surveillance system.
3. The author’s recommendations ought to be specific to the gaps identified in their evaluation or emanating from the discussion of the results. Specific and targeted.
