# Supplementary material for: Maternal death surveillance and response system evaluation in Makonde District, Zimbabwe, 2021
Source: PLoS One. 2024 Jul 1;19(7):e0301929. doi: 10.1371/journal.pone.0301929 (PMC11216583; doi:10.1371/journal.pone.0301929)
Supplement: Attachment — Submitted filename: RESPONSE TO REVIEWERS.docx [file pone.0301929.s004.docx]

Dear Editor/Reviewers

RE: Responses to comments raised in our submitted manuscript **‘Maternal death surveillance and response system evaluation in Makonde District, Zimbabwe, 2021’**

Thank you for reviewing our manuscript and for the constructive comments. Please find below our responses to the comments.

| **Section** | **Editor’s comments** | **Response** |
| --- | --- | --- |
| Introduction | 1. Your introductory statement “…with 810 women dying every day from preventable causes related to pregnancy” refers to figure as of 2017, five years behind. This (2017) should be referenced in the statement. Alternatively, the authors should revise the statement to report the current figures of maternal mortality globally and in sub-Saharan Africa and then in Zimbabwe. | We have revised the statement and put current figures recorded by WHO for 2020. The current figures for maternal mortality are included in the introduction already. They have been rearranged. |
|  | 2. The statement “Globally the maternal mortality ratio is declining however the average rate of decline is not fast enough to achieve SDG target of 70 maternal deaths per 100000 live births” is similar to the statement “The United Nations Sustainable Development Goal target 3.1 aims at reducing global maternal mortality ratio to less than 70 per 100 000 live births by 2030…” and conveys the same meaning. I suggest you consider merging the two. | Thank you, we have merged the statements. |
|  | 3. The authors referenced “the Centre for Disease Control” which collaborated with the World Health Organization. Could you be specific about the particular Centre for Disease Control? We have so many Centres for Disease Control who could partner WHO to implement programmes in LMIC. | It is the US CDC Division of Reproductive Health. I have added it to the manuscript. |
|  | 4. The authors explained that the “Maternal Death Surveillance and Response System (MDSR)” is what is partially being evaluated in Makonde District. Perhaps, the authors could be much clearer on the motivation for choosing the Makonde District. Although the authors explained that 62% of maternal death were notified and audited which presumably is low for them, this figure is left standing alone could rather be an impressive figure if compared contextually. Perhaps, it could be compared with the figures of other districts who implemented the “MDSR” to show a compelling reason for wanting to know the situation in the Makonde District in particular. | The highest number of maternal deaths was recorded in Makonde District. The provincial referral hospital is in Makonde District where most of the complicated maternity cases are referred to. The remaining six districts in the province achieved 100% notification and auditing of maternal deaths save for one district which achieved 80% with the second highest number of maternal deaths.  Moreover, Makonde recorded the highest number of maternal deaths versus the highest deliveries. |
|  | 5. Also, note that “MDSR” used as an abbreviation in the manuscript does not exactly represent the “Maternal Death Surveillance and Response System (MDSR). Probably MDSRS. Please check and revise appropriately. | Thank you for the comment. We have researched and it is abbreviated as MDSR. The word “system” is not part of the abbreviation. We have taken note of it in the manuscript. It is written as MDSR system |
|  | 6. **General Comment** - The introductory aspects is generally scanty and does not address the topic area in literature, rational for the choice of study area and purpose of study, adequately. | We have added flesh to the introduction capturing some literature, rationale for choice of study area and purpose of study. |
| Study setting | 1. The authors gave statistics of antenatal care attendance in the district of study to include one visit etc. Again, we are not told what percentage made 8+ visits and if none, indicate so to put the reference data into proper perspective, especially as the WHO was part of the implementation of the MDSR. | Thank you we have included the statistics. None had attended eight or more ante-natal care visits stipulated by WHO. |
|  | 2. The authors also left out health facility delivery percentages from the Makonde District, although they attempted to explain that health facilities existed widely with capabilities and with one referral centre – the Chinhoyi Provincial Hospital. Please add facility delivery figures of the district. It will be useful in your discussion | We have added the delivery percentages of the different facilities in the study setting section.  Number of deliveries and maternal deaths in Makonde district health facilities offering maternity delivery services are as follows;  Chinhoyi provincial hospital 6920 deliveries and 22 maternal deaths  Makonde Christian hospital 1915 and 0 deaths  Mtala council clinic 537 and 1 death  Kanyaga Rural health Centre 408 and 0 deaths  St Ruperts mission hospital 314 and 0 deaths  Umboe rural health centre 314 and 0 deaths  Manyamba rural health centre 195 and 1 death  Sadoma council clinic 193 and 0 deaths  Shackleton council clinic 193 and 0 deaths  Kenzamba rural health centre 173 and 0 deaths  Kamhonde rural health centre 171 and 0 deaths  Green Valley rural health centre 163 and 0 deaths  Chitambo private hospital 155 and 0 deaths  River ranch rural health centre 139 and 0 deaths  Municipal urban clinics 26 and 0 deaths  Data has been presented as at table in the manuscript |
| **Maternal Death Surveillance and Response System in Zimbabwe** | 1. **The statement** “…. health facilities and communities should include zero reporting in their routine reports” is not clear. Perhaps, you could explain what you mean by zero reporting | Zero reporting means reporting of the absence of an event under surveillance for the review period. A report with a zero for maternal deaths is thus submitted in this instance. We have explained in the manuscript. |
|  | 2. *Figure 1: Flow of information in a Maternal Death Surveillance and Response System in Zimbabwe* is a good representation of information flow relative to the surveillance system. However, Mission Hospital/ Local Authorities is left standing alone with no link to source of information as though it is the final generator of information. It is not clear if the diagram is adopted or adapted or the original creation of the authors. Perhaps the authors could consider creating a link of information from the community to the mission hospital/local authority, since the community is the original and last source of maternal data. | The flowchart now shows the connection between the community and mission and local council clinics. |
|  | 3. Also, I don’t find an in-text reference to Figure 1. which makes the figure appear not useful in the manuscript, but that is not the case. Please reference the figure in the write up appropriately. | **Thank you I have referenced Figure 1 in text.** The flow of information in a Maternal Death Surveillance and Response system in Zimbabwe is shown in Figure 1. |
| Study design | 1. “The updated CDC guidelines for 163 Surveillance System Evaluation” … was the updated version utilized in the Makonde District surveillance, otherwise, the authors may have to explain why they chose the updated version of CDC guidelines. | The surveillance system in Zimbabwe as a whole is premised on satisfying attributes that are stipulated in the CDC guidelines. Any evaluation therefore seeks to check whether these attributes are being met. That is why we used the updated guidelines to evaluate the system in Makonde District. |
|  | 2. What is “T5” registers? | It is a register which summarises all health events captured in a month. However, we have removed the term T5 and described the type of register in the study design. |
| Sample size and data collection | 1. **“A minimum sample size of 61 was calculated”.** This does not marry well with “we interviewed five doctors and forty nurses from the provincial hospital, two doctors and six nurses from the rural hospital, one nurse and one doctor from the private maternity clinic, ten nurses from the urban clinics and fourteen nurses from the rural clinics”. Clarity is necessary. 2. Your sample calculation of the mortality record for desk top review must stand out clear from your choice of health care professionals for interviews. | We calculated a minimum sample size of 61. Looking at the establishment of doctors and nurses in post, 8% were doctors and 92% were nurses which translates to interviewing at least 5 doctors and 56 nurses. We therefore managed to interview 8 doctors and 71 nurses over the period we carried out the study. Coming down to the facilities we also used proportions based on the establishment. From the provincial hospital we calculated at 31 nurses and 4 doctors, 14 nurses from the rural clinics, 6 nurses and 1 doctor from the rural hospitals and 5 nurses from the urban clinics. We managed to reach the samples sizes calculated for each facility however additional doctors and nurses were obtained from the referral hospital where most of the deaths and discrepancies were observed.  All mortality records over the study period were reviewed. |
|  | 3. Could the authors share the data collection tools? | Yes |
| Ethical consideration | 1. Perhaps the authors could quote the approval code or number from the Mashonaland West Provincial Ethics Committee. | Thank you. The approval number is JFREC-NAC/3/22 |
| Results | 1. Only 41% were midwives, although a large portion of the health professionals interviewed were nurses. The expectation is that midwives should be the main target group for interview in matters of maternal deaths and audit. The authors need to explain further why this was not the case. Perhaps numbers and availability and willingness, to add meaning to the results. | We have given reasons why we had only 41% midwives among the interviewed nurses in the discussion. The major reason was high staff turnover. |
|  | 2. The writing of the results should be re-organised to give a good flow. For example, “Overall, 61 (77.2%) of the 248 healthcare workers displayed good knowledge of the MDSR system. Table 3 shows the results” should be the opening paragraph of that section and followed by other relevant portions of the results which the authors intend to communicate rather than make this statement in the ending paragraphs. | We have rearranged the writing of the results. Thank you. |
|  | 3. “Assuming a nurse’s salary of $400 USD and a distance of 120 km of the furthest health facility to the district hospital” what is the need for introducing a nurse’s salary in the cost component of the results write up? It appears tangential to the study discourse. In any case, the entire idea of cost does not add value to the study objective. It can be removed without a significant damage to the study results. | We agree with the reviewer and we have removed cost component in the results section |
| Discussion | 1. Generally good | Thank you |
|  | 2. The authors made a statement that “Healthcare workers in Makonde District presumed the MDSR system to be useful as they utilised the information generated from audits to improve maternal health service delivery and monitor maternal mortality” yet, the district doesn’t seem to achieve what is expected. Could there be some conflicting reasons??? This needs to come out clearly in the discussion. | We have indicated in the discussion that to some extent the system was useful as information generated from the system was utilised in the district to improve future outcomes however they were failing to achieve the expected and missing some opportunities that would be useful in improving the system. |
| Limitation of the study | 1. Although the study is useful, it is limited in many ways. For example, not targeting midwives more in the study, given they are the professional grouping mandated to handle pregnant women limits the validity of information obtained from the health workers. This has to be stated in the manuscript. | Thank you for the guidance we have included the limitation. |
| Conclusion and recommendations | 1. Your results show 87.3% know the importance of the tool, 91% know the timeliness importance, 97% thinks the system is useful, 88.6% are willing to continue with the surveillance system yet **“lack of motivation” and “training gap”** appeared in your conclusion? Readers will not understand this. The authors need to revise their conclusion based on the study results. | We have edited the conclusion not to include lack of motivation and training gaps. However recommending on going on job training and refreshers especially with the high staff turnover and having general nurses who are not midwives working in the maternity wards. |
|  | 2. The authors rather gave general recommendation including training requirement of staff and also recommended the need for ambulance services to be effectively provided. I don’t find these as borne out of the study results/discussion, especially as the surveillance process did not involve ambulance system and the results suggest the staff had good knowledge of the surveillance system. | Thank you, we have revised the recommendations section and removed general training and included training of more midwives. |
|  | 3. The author’s recommendations ought to be specific to the gaps identified in their evaluation or emanating from the discussion of the results. Specific and targeted. | We have revised the recommendations section.  We have focused my recommendations on the following findings;   1. Fewer midwives and high staff turn over 2. Poor data management. 3. Timeliness of notification and audits |
| Reviewer 2 comments | There is profound similarity in the aim, study design and methodology of the submitted manuscript with the above publication. You may also include additional comments for the author, including concerns about dual publication, research ethics, or publication ethics. (Please upload your review as an attachment if it exceeds 20,000 characters) | We have reworked the study design and methodology in the manuscript to exclude similarities. |
