# Supplementary material for: Maternal death surveillance and response system evaluation in Makonde District, Zimbabwe, 2021
Source: PLoS One. 2024 Jul 1;19(7):e0301929. doi: 10.1371/journal.pone.0301929 (PMC11216583; doi:10.1371/journal.pone.0301929)
Supplement: Attachment — Submitted filename: Response to Reviewers.docx [file pone.0301929.s005.docx]

Responses to comments raised in our submitted manuscript **‘Maternal death surveillance and response system evaluation in Makonde District, Zimbabwe, 2021’**

Thank you for reviewing our manuscript and for the constructive comments. Please find below our responses to the comments.

| **Section** | **Editor’s comments** | **Response** |
| --- | --- | --- |
| General | This manuscript should be proofread by a native English speaker | I have given it to two colleagues who have assisted with grammar, thank you. |
| Abstract | I noted the cost of notifying and investigating a single death was USD$65.18. Please clarify to your readers its relevance as it was ONLY mentioned in the abstract and not elsewhere. | Thank you for bringing this up. I have removed the cost aspect in the abstract. It was an omission, I deleted the cost aspect in the main manuscript as advised in the previous set of comments and forgot to delete the aspect in the abstract. |
| General | The manuscript needs to formatted in accordance journal's manuscript style. | I have formatted the manuscript. |
