# Supplementary material for: Maternal death surveillance and response system evaluation in Makonde District, Zimbabwe, 2021
Source: PLoS One. 2024 Jul 1;19(7):e0301929. doi: 10.1371/journal.pone.0301929 (PMC11216583; doi:10.1371/journal.pone.0301929)
Supplement: Attachment — Submitted filename: Response to Reviewers.docx [file pone.0301929.s006.docx]

Responses to comments raised in our submitted manuscript **‘Maternal death surveillance and response system evaluation in Makonde District, Zimbabwe, 2021’**

Thank you for reviewing our manuscript and for the constructive comments. Please find below our responses to the comments.

| **Section** | **Editor’s comments** | **Response** |
| --- | --- | --- |
| General | Align all the lines in the entire document. | I have done so, thank you. |
| General | Table contents and labels should be unbolded. | Thank you, I have unbolded the labels and contents |
| General | Examine the style of referring | I have revisited and added missing information on some references and edited some |
